# Case Report: Heterogeneity of Resistance Mechanisms in Different Lesions Co-Mediate Acquired Resistance to First-Line Icotinib in EGFR Mutant Non-Small Cell Lung Cancer

**DOI:** 10.3389/fmed.2022.906364

**Published:** 2022-07-07

**Authors:** Zhicong Liu, Hui Dong, Wenyan Chen, Bin Wang, Dongxiang Ji, Wei Zhang, Xuefei Shi, Xueren Feng

**Affiliations:** ^1^Department of Respiratory Medicine, Huzhou Central Hospital, Affiliated Central Hospital Huzhou University, Huzhou, China; ^2^Department of Respiratory Medicine, Huzhou Hospital, Zhejiang University School of Medicine, Huzhou, China

**Keywords:** lung adenocarcinoma, resistance mechanisms, ALK rearrangement, EGFR exon 20 T790M, combination therapy, case report

## Abstract

Epidermal growth factor receptor (EGFR)-activating mutations are major oncogenic mechanisms in non-small cell lung cancer (NSCLC). Most patients with NSCLC with EGFR mutations benefit from targeted therapy with EGFR- tyrosine kinase inhibitors (TKIs). One of the main limitations of targeted therapy is that the tumor response is not durable, with the inevitable development of drug resistance. Previous studies demonstrated that the potential resistance mechanisms are diverse, including the presence of EGFR T790M, *MET* amplification, mesenchymal transformation, and anaplastic lymphoma kinase (*ALK*) rearrangement. The patient in our report was diagnosed with stage IA lung adenocarcinoma harboring the EGFR L858R mutation and underwent radical surgery. The patient received icotinib for 12 months after recurrence. Subsequent molecular analysis of the left pleural effusion indicated that *LCLAT1-ALK* fusion might be an underlying mechanism contributing to the acquired resistance to icotinib. Ensartinib was prescribed, but the lesion in the right lung continued to progress. Hence, a re-biopsy and molecular analysis of lesions in the right lung was performed to solve this problem. In contrast to the left pleural effusion, EGFR exon 20 T790M might have mediated the acquired resistance in lesions in the right lung of this patient. The combination of osimertinib and ensartinib has achieved a rapid partial response until now. The complexity and heterogeneity in our case may provide new insights into the resistance mechanisms of targeted therapy.

## Background

Lung cancer is one of the most frequently diagnosed cancers and the leading cause of cancer-related death worldwide (1). Historically, systemic cytotoxic chemotherapy has been the predominant treatment for advanced-stage lung cancer (2). However, lung cancer is a heterogeneous disease requiring personalized treatment. The development of molecular detection technologies has allowed the identification of multiple and potentially targetable oncogene drivers and personalized targeted therapies for lung cancer (3). Some well-established targets include epidermal growth factor receptor (*EGFR*), anaplastic lymphoma kinase (*ALK*), and ROS proto-oncogene 1 (*ROS1*) (4, 5). Over the past two decades, several EGFR inhibitors have been developed. For example, the first-, second-, and third-generation EGFR inhibitors, icotinib, afatinib, and osimertinib, have been developed to treat mutated EGFR (mtEGFR) non-small cell lung cancer (NSCLC) (6, 7). Despite excellent response rates to these drugs, patients invariably experience disease progression due to the emergence of drug-resistant tumors, usually within 9–14 months, which is a major hurdle in EGFR tyrosine kinase inhibitor (TKI) therapy (8). However, the mechanism underlying acquired drug resistance in patients treated with EGFR-TKIs remains unclear. In this study, we report a case of a patient with different drug resistance-associated mutations (EGFR exon 20 T790M and *LCLAT1-ALK* fusion), which demonstrated the related resistance mechanisms after first-line icotinib treatment to be highly heterogeneous.

## Case Presentation

**Figure 1:**
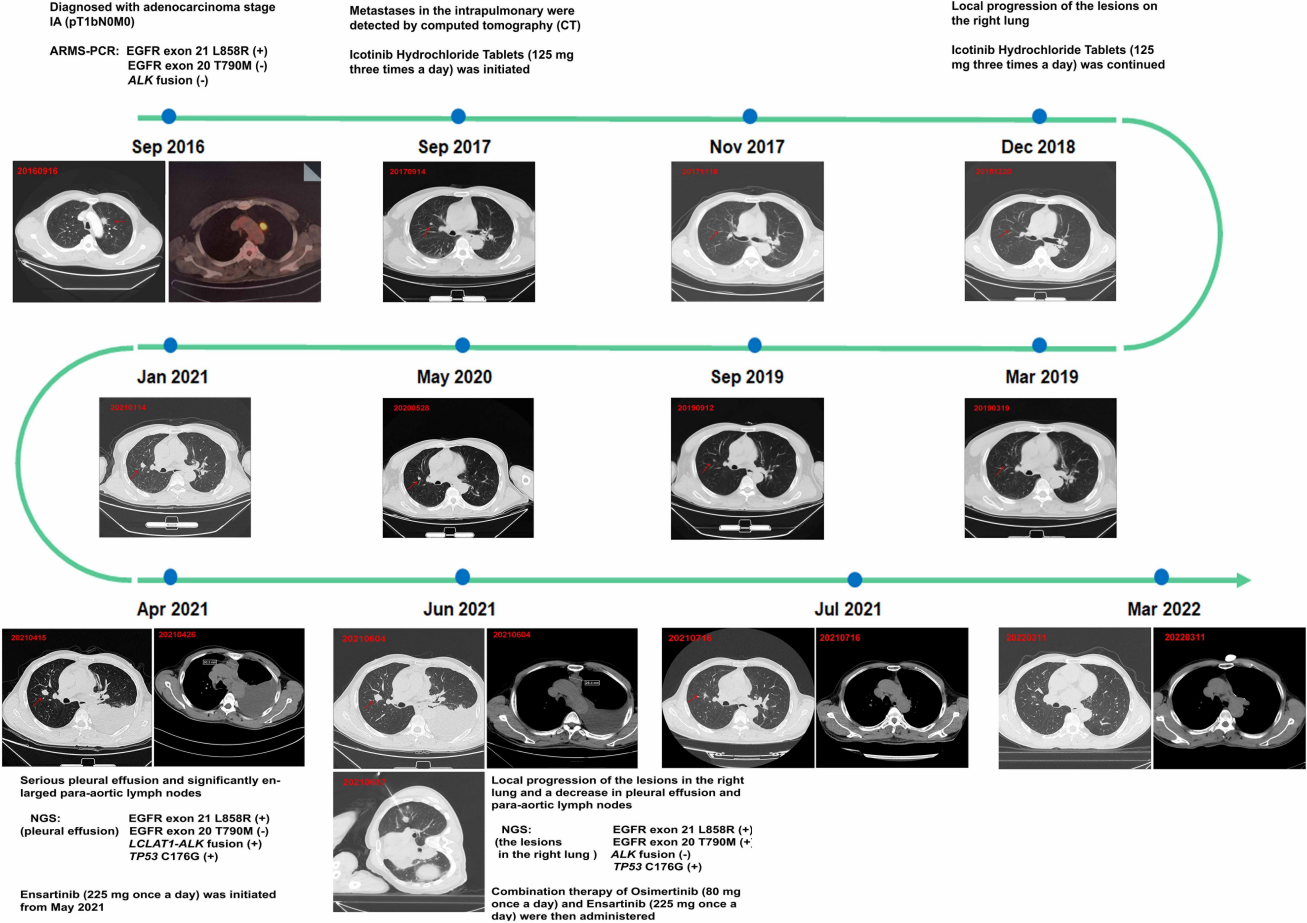
Treatment course with corresponding computed tomography (CT) scans. This figure shows the timeline of progression after 43 months under the treatment with only icotinib; the patient achieved a satisfactory exceptional response by treatment with osimertinib plus ensartinib.

**Figure 2:**
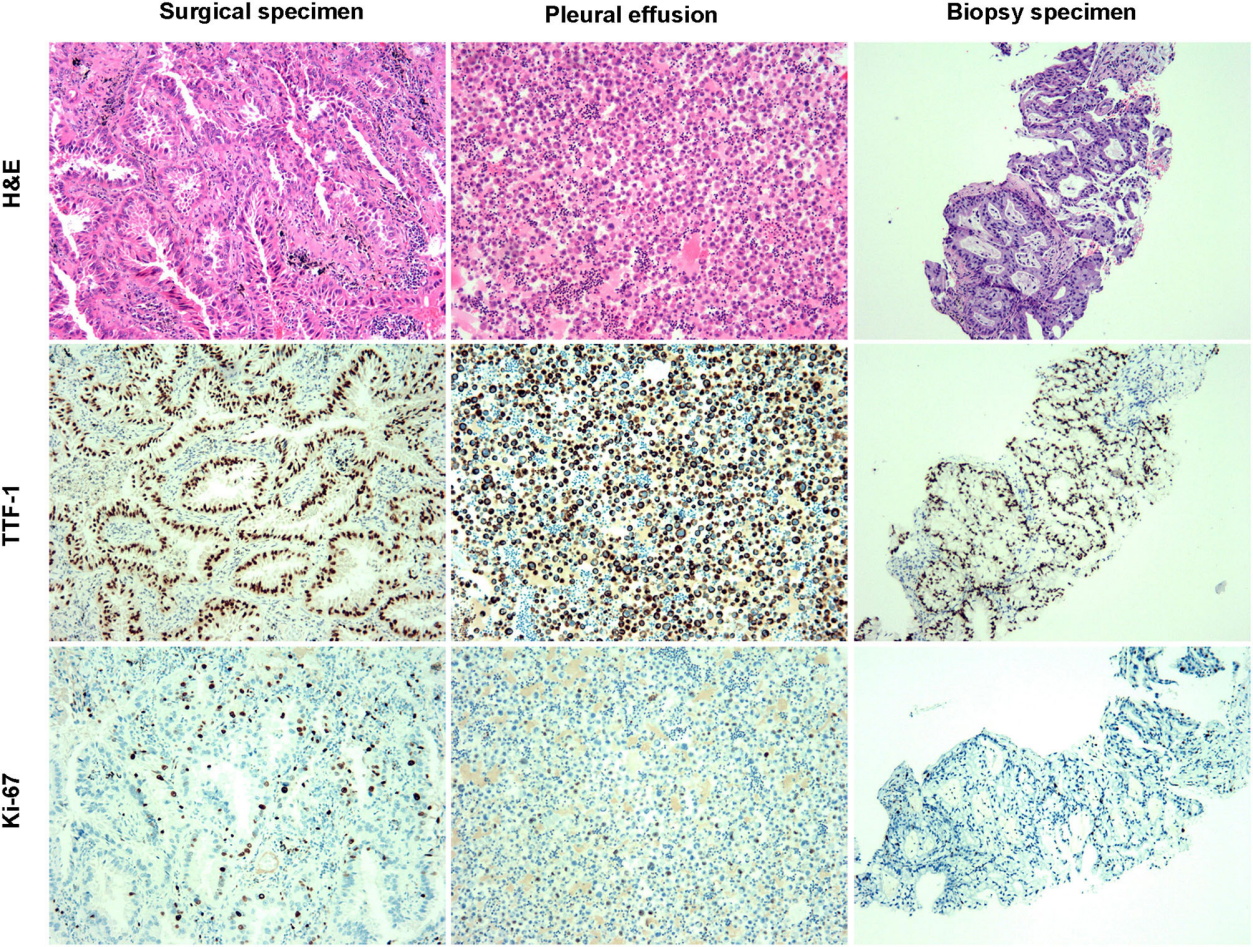
The pathological diagnosis of specimens was lung adenocarcinoma. H and E staining and immunohistochemistry staining of surgical, pleural effusion, and biopsy specimens.

A 64-year-old man with no smoking history and no history of cancer presented to our hospital in September 2016 with a primary tumor (1.5 × 1.5 cm) in his upper left lung (Figure 1). A positron emission tomography/computed tomography (PET/CT) scan revealed a mass in the upper left lung with intense uptake of (18F) fluorodeoxyglucose and no distance metastasis. Postoperatively, the patient was diagnosed with adenocarcinoma stage IA (pT1bN0M0). The immunohistochemical staining is shown in Figure 2. Amplification refractory mutation system polymerase chain reaction (EGFR/ALK/ROS1) of the resected tissue identified EGFR exon 21 L858R, and negativity for *ALK* fusion (Table 1). After 12 months, intrapulmonary metastases were detected by computed tomography (CT). Therefore, treatment with icotinib hydrochloride tablets (125 mg three times daily) was initiated in September 2017. The patient experienced a rapid partial response (PR) according to the Response Evaluation Criteria in Solid Tumors (RECIST).

**Table 1:** Molecular detection during treatment of the patient in the study.

**Time of sampling**	**Samples**	**Methods**	**Mutation style**	**Allele frequency (AF)**
Sep 2016	Tumor tissue (left)	ARMS-PCR	EGFR exon 21 L858R	–
Apr 2021	Pleural effusion (left)	NGS (48-gene panel*)	EGFR exon 21 L858R *LCLAT1* exon 6*-ALK* exon 20 fusion TP53 C176G	27% 2% 16%
	Pleural effusion (left)	ARMS-PCR	EGFR exon 21 L858R ALK exon 20 fusion	– –
	Pleural effusion (left)	IHC	ALK (+)	
Jun 2021	Tumor tissue (right)	NGS (48-gene panel*)	EGFR exon 21 L858R EGFR exon 20 T790M mutation TP53 C176G	6% 10% 6%

*NGS, next-generation sequencing.*

*48-gene panel including AKTI, ALK, APC, BRAF, BRCA1, BRCA2, CCN, CD274(PD-L1), CDK4, CDK6, C, DKN2A, CDKN28, EGFR, ER8B2(HER2), ERBB4, ESR1, FGFR1, FGFR2, FGFR3, FGFR4, KIT, KRAS, MAP2KI(MEKD), MET, MLH1, MSH2, MSH6, MTOR, NF1, NFE2L2(NRF2), NRAS, NTRK1, NTRK2, NTRK3, PALB2, PDCD1(PD-1), PDGFRA, PDGFRB, PIK3CA, PMS2, PTEN, RB1, RET, ROS1, STK11, TP53, TSC1, and TSC2.

The timeline of the disease progression is shown in Figure 1. After 15 months of icotinib treatment, a CT scan of the patient revealed progressive disease (PD) (Figure 1). Due to the local progression of the lesions in the right lung, icotinib treatment was continued until April 2021. The patient experienced serious pleural effusion on the left side and significantly enlarged para-aortic lymph nodes (6, 30.3 mm) (Figure 1). Pleural drainage was performed to alleviate the clinical symptoms, and next-generation sequencing (48-gene panel) of the pleural effusion was conducted to explore the mechanism of icotinib resistance. The results revealed an *LCLAT1-ALK* fusion and TP53 C176G in addition to EGFR exon 21 L858R (Table 1). Meanwhile, we verified the result via ARMS-PCR analysis and found that *ALK* exon 20 fusion-positive. We also detect the ALK protein level by IHC (ALK D5F3). Consequently, the patient started ensartinib (225 mg one time daily) as the next treatment in May 2021, as ensatinib has fewer adverse reactions than ceratinib. After 1 month, chest CT revealed local progression of the lesions in the right lung, as well as a decrease in pleural effusion and para-aortic lymph nodes (6, 28.4 mm). Given the possibility of different drug resistance mechanisms in different lesions, a biopsy specimen was obtained by CT-guided fine-needle aspiration (FNA). Next-generation sequencing (48-gene panel) of the tissue samples obtained from the lesions on the right lung in June 2021 revealed EGFR exon 20 T790M mutation, TP53 C176G, and EGFR exon 21 L858R, with no *ALK* fusion (Table 1). Hence, combination therapy with osimertinib (80 mg one time daily) and ensartinib (225 mg one time daily) was administered. One month later, a CT scan revealed considerable reductions in the lesion sizes in the right lung, para-aortic lymph nodes, and pleural effusion. Regular follow-up chest CT indicated that the lesion was stable through March 2022 (Figure 1). During the treatment with combination therapy, the patient developed a local rash and transient elevation of his serum creatine kinase level, which required no special treatment.

The patient provided written informed consent for the publication of this case.

## Discussion and Conclusion

The mechanism of acquired drug resistance in patients treated with EGFR-TKIs is currently under investigation. The evolutionary pressure acting on cancer cells *via* spatial and temporal clonal selection, combined with the random acquisition of genetic mutations, contributes to drug resistance (9). The most commonly observed acquired resistance mechanism is the acquisition or outgrowth of the T790M mutation (exon 20 of EGFR), which accounts for approximately 60% of patients receiving gefitinib, erlotinib, or icotinib (10). Osimertinib is an irreversible third-generation EGFR TKI administered for the first-line treatment of common sensitive *EGFR* mutations, or for second-line treatment of acquired resistance, to first-generation EGFR-TKIs by selectively targeting the T790M mutation (11). The results of the AURA3 (NCT02151981) phase III trial revealed that osimertinib significantly prolonged progression-free survival (PFS) compared to platinum and pemetrexed chemotherapy (10.1 months vs. 4.4 months, *P* < .001) in patients with the T790M resistance mutation after the failure of prior TKIs (12). In addition to *EGFR* exon 20 T790M, several gene fusions involving driver oncogenes are rarely acquired resistance mechanisms in patients treated with first-generation EGFR-TKIs. A previous study reported that only 13% of patients with NSCLC-harboring-EGFR mutations acquired EGFR TKI resistance mediated by emerging *ALK* rearrangements (13). Hu et al. (14) reported a patient with lung adenocarcinoma who displayed alternate drug resistance changes between *EGFR* and *ALK* after gefitinib resistance. Similarly, Hou et al. (15) summarized previously reported cases, in which *ALK* rearrangement or fusion was a rare but critical resistance mechanism to osimertinib. The most common alteration in the *ALK* gene is chromosomal rearrangements, such as *EML4* (chromosome 2)-*ALK* (chromosome 2) (16). Xia et al. (17) revealed complex *ALK* gene fusion by targeted-capture DNA-based NGS, RNA-based NGS, RT-PCR, IHC, and FISH. In 343 samples of existing *ALK* fusions, they identified that intron 1 of LCLAT1 joined between the intron 13 of *EML4* and the intron 19 of *ALK*. In our case, we identified a novel *ALK* gene fusion, *LCLAT1* exon6*-ALK* exon 20 by DNA-based NGS. *LCLAT1* exon6 has not been previously reported as a partner gene of ALK. Unfortunately, due to the limitation of specimens, we could not perform RNA-based NGS to explore the RNA transcript of *LCLAT1* exon6*-ALK* exon 20, which is a limitation of our case. According to the result of DNA-based NGS, IHC, and the effect of ensatinib, we preliminarily speculated that the RNA transcript might be LCLAT1-ALK (exon6: exon20).

The increasing understanding of tumor heterogeneity has revealed that specific resistance mechanisms may occur in different clones. A deeper understanding of the complexity of EGFR-TKI resistance will help to guide clinical decisions. In our case, two different acquired resistance mechanisms occurred in two lesions following the development of icotinib resistance. This phenomenon reflects the heterogeneity of the original tumor. Therefore, when targeted treatment fails, re-biopsy and molecular analysis are considered standard procedures to solve this issue. In our report, the patient achieved a satisfactory response after treatment with osimertinib plus ensartinib. Patients with EGFR/ALK co-alterations may benefit from combined treatment with both TKIs, including long-term survival (18).

## Data Availability Statement

The original contributions presented in the study are included in the article/supplementary material, further inquiries can be directed to the corresponding authors.

## Ethics Statement

Written informed consent was obtained from the individual(s) for the publication of any potentially identifiable images or data included in this article.

## Author Contributions

WC, WZ, and HD drafted the manuscript. XF, ZL, and BW treated the patient. XS and DJ retrieve the related literature. All authors contributed to the article and approved the submitted version.

## Funding

This work was supported by grants from the Project of Zhejiang Basic Public Benefit Research of Zhejiang Province (No. LGD21H010001 to ZL, No. LGF21H160003 to BW). The foundation supported gene mutational analysis.

## Conflict of Interest

The authors declare that the research was conducted in the absence of any commercial or financial relationships that could be construed as a potential conflict of interest.

## Publisher's Note

All claims expressed in this article are solely those of the authors and do not necessarily represent those of their affiliated organizations, or those of the publisher, the editors and the reviewers. Any product that may be evaluated in this article, or claim that may be made by its manufacturer, is not guaranteed or endorsed by the publisher.
